# The Initial Immune Reaction to a New Tumor Antigen Is Always Stimulatory and Probably Necessary for the Tumor's Growth

**DOI:** 10.1155/2010/851728

**Published:** 2010-07-27

**Authors:** Richmond T. Prehn

**Affiliations:** Department of Pathology, School of Medicine, University of Washington, 5433 South Hudson St., Seattle, WA 90118, USA

## Abstract

All nascent neoplasms probably elicit at least a weak immune reaction. However, the initial effect of the weak immune reaction on a nascent tumor is always stimulatory rather than inhibitory to tumor growth, assuming only that exposure to the tumor antigens did not antedate the initiation of the neoplasm (as may occur in some virally induced tumors). This conclusion derives from the observation that the relationship between the magnitude of an adaptive immune reaction and tumor growth is not linear but varies such that while large quantities of antitumor immune reactants tend to inhibit tumor growth, smaller quantities of the same reactants are, for unknown reasons, stimulatory. Any immune reaction must presumably be small before it can become large; hence the initial reaction to the first presentation of a tumor antigen must always be small and in the stimulatory portion of this nonlinear relationship. In mouse-skin carcinogenesis experiments it was found that premalignant papillomas were variously immunogenic, but that the carcinomas that arose in them were, presumably because of induced immune tolerance, nonimmunogenic in the animal of origin.

## 1. Introduction

The immune surveillance hypothesis, championed in the 60s by Burnet [1] to explain why many cancers, although part of the self, are nonetheless immunogenic, has had a disputatious history. One of the earliest arguments in favor of the surveillance hypothesis was the observation that immunodepression increased the incidence of certain human cancers, especially those of the skin and of the lymph system [2]. However, Stutman, in a comprehensive survey, could find no evidence that immunodepression affected carcinogenesis [3]. More recently, Schreiber and Podack have reopened the question by publishing an analysis of the validity of the immune surveillance hypothesis in the case of methylcholanthrene-induced carcinogenesis [4]. Their conclusion was, in essence, that immunodepression often increased tumor growth and/or incidence, thus supporting the existence of anticancer immune surveillance.

## 2. Two Illustrative Experiments

The late Dr. Marc Lappé and I published a paper apparently showing the role of immune surveillance during chemically induced skin carcinogenesis [5]. We based these studies upon a system in which a suboncogenic dosage of 3-methylcholanthrene (MCA) was applied as an “initiator” to the backs of BALB/c mice. After a short interval, the initiated skin was “promoted” by orthotopic transplantation to syngeneic mice whose immune capacities had been modified by various means; the transplanted skins were then observed over time for the development of skin papillomas (paps) and carcinomas. All carcinomas arose within previously identified paps on the skin grafts and were easily identified by gross examination [5]. Transplantation proved to be an efficient promoter and was used to avoid, as far as possible, systemic immunodepressive effects on the host animals by the initiating MCA.

Lappé's work suggested that the host's immune capacity can modify both the incidence and the duration of MCA-induced skin paps, albeit primarily the duration. Most paps ultimately regressed. At the time the work was performed, it was assumed that the longer duration of paps and the higher incidence of carcinomas in the immunodepressed animals, in the Lappé and similar experiments, were supportive of the immune surveillance hypothesis [5].

Subsequently, another student of mine performed experiments very similar to those of Lappé, but with a slightly different objective [6]. The question asked by Andrews was whether or not paps could regress in the complete absence of immunity (or in as nearly complete absence as could technically be obtained)? The experimental protocol was modified as follows: as a further test of the efficacy of the imposed immunodepression, “promotion” was achieved by transplanting the MCA-initiated skin to allogeneic rather than to isogenic recipients. Prior to transplantation, the hosts were immunodepressed by thymectomy and x-radiation as well as by weekly injections of antilymphocyte serum. Although in one experiment many animals became ill, in most experiments survival appeared to be normal, and the allografts survived indefinitely. By several different additional tests, no residual immune capability was detectable [6].

 Andrews' data showed that, in the absence of detectable immune capacity in the hosts, MCA-induced papillomas occurred and persisted for varying lengths of time before most of them regressed; to our surprise, none progressed to carcinoma [6].

The Lappé and Andrews data seem discordant in that Lappé found that a moderate immunodepression increased the carcinoma incidence; Andrews, in contrast, found that an even greater degree of immunodepression vastly reduced the incidence of carcinomas. How can the Lappé and Andrews' data sets be reconciled?

## 3. The Hormetic Nature of the Immune Response Curve

**Figure 1:**
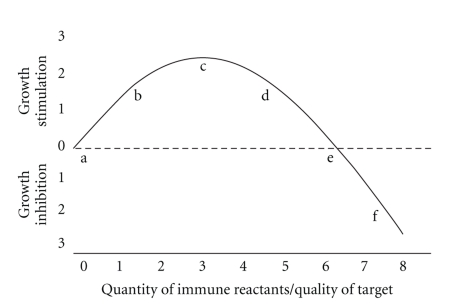
Immune response curve (IRC). Idealized curve derived from data in [7] showing the hormetic shape of the curve titrating the quantity of immune reactants against the effect on tumor growth. Letters and numerals are arbitrary aids to discussion.

The key to this seeming discrepancy probably lies in the fact that the two sets of experiments were done at very different points on the titration curve of the immune response. This titration curve was almost certainly not linear. In 1972, I showed that the effect of quantitative variations of adaptive immunity on tumor growth followed an immune response curve (IRC) suggestive of hormesis [7] (see Figure 1).

This same basic observation has been made many times and in many different experimental systems; low quantitative levels of immunity tend to stimulate both carcinogenesis and the growths of tumor implants while larger amounts of apparently the same immunological reactants are inhibitory to neoplastic growths [8]. Thus, when a tumor grows better, either faster or in greater frequency as a result of immunodepression, one often cannot be sure whether the result was caused by a lessened inhibition or by a greater stimulation of the growth of the target neoplasm. The correct interpretation depends upon knowing the often unknowable; knowing where on the nonlinear quantitative scale of immune effects the reaction actually resides.

Since no malignancies occurred in the near absence of an immune reaction, the work of Andrews suggests strongly that, in the system he explored, a greater stimulatory effect of immunity would have been helpful and perhaps even necessary for papilloma progression to carcinoma; this is the expected result of immune reactions falling far to the left of “c” on the IRC in Figure 1. Also, it may perhaps be possible to postulate that papilloma regression may have been, at least in part, a consequence of insufficient immune stimulation It thus becomes reasonable to ask whether the longer-lasting paps in the immunodepressed mice in the Lappé work were also a consequence of greater immune stimulation rather than a relative paucity of immune inhibition. Lappé definitely showed that immunodepression increased the incidence of MCA-induced skin carcinomas while an artificially increased immune capacity was associated with fewer carcinomas [5]; thus, most of the Lappé paps must have occurred in an area of the titration curve where a lessened or an increased host immune capacity would result in greater or lesser papilloma incidence respectively, that is, somewhere to the right of  “d” on the IRC (Figure 1). Whether the paps fell to the right or to the left of “e”, that is, whether the immunity to the paps was in the stimulatory or the inhibitory range, remains unknown by this analysis, but I will argue subsequently that it was probably in the stimulatory portion of the curve.

One caveat must be noted at this point. As the immune reaction is presently understood, the fact that Andrews used allogeneic transplantation for “promotion” while Lappé used syngeneic should not have been a significant difference; however, if there was a difference in the promotional effects of the two procedures, the papillomas in the two experiments might not have been exactly comparable.

In the Lappé experiment, it is apparent that the papillomas were immunogenic in as much as their longevity was markedly influenced by the imposed alteration of the hosts' immune capacities. However, the immune capacities of the host animals had little or no influence upon the growths of the carcinomas that resulted from the progression of the immunogenic paps; as already noted, a quite constant percentage of surviving paps became carcinomatous in subsequent successive intervals of observation. It therefore follows that in the Lappé experiments, overt tumor progression coincided with the development of a marked degree of immunological tolerance to the tumor antigens.

It would seem logical that any newly engendered immune reaction begins small and only with time might become sufficiently robust to inhibit, rather than stimulate, tumor growth; the immune reaction to any tumor antigen must, given the essential correctness of the IRC, begin at the extreme left end of that curve; thus the initial immune reaction to newly presented tumor antigens is necessarily always stimulatory. 

The prolongation of the longevity of paps in the Lappé immunodepressed mice, and in all similar oncogenesis experiments in which immunodepression results in more tumor growth, may often be caused by immune stimulation of the tumors rather than by the reduction of an assumed tumor inhibition. Whenever immunodepression results in fewer tumors and/or less tumor growth, the system, with the immunodepression being absent, was probably near or to the left of “c” and was moved even further to the left by the reduced immune reaction; in contrast, whenever immunodepression results in more tumors and/or more tumor growth, the system, with the immunodepression being absent, would have fallen somewhere to the right of “c” and be moved further toward “c” by the immunodepression. The fact that the initial immune reaction to a tumor is necessarily stimulatory to tumor growth does not imply that the reaction might not, in some cases, eventually grow to inhibitory levels (to the right of “e”) and, in such cases, immunodepression would also result in higher tumor incidences.

Stutman, in his already mentioned survey concerning the lack of effect on oncogenesis by immunodepression [3], could find no evidence of either immune surveillance or of immune stimulation. I suggest that this result would be expected if most carcinogenesis experiments actually fall near “c” on the ICR; in some cases, oncogenesis would be relatively inhibited and in others relatively stimulated by further immunodepression of the already weak immune response to the incipient tumor. In some cases, variation around “c” among the individual tumor antigenicites might well result in examples of both relative inhibition and relative stimulation of tumor growth by immunodepression within a single experiment, thus producing a null effect.

## 4. Tumor Antigens

Cancers evoked by chemical carcinogens and by oncogenic viruses usually contain strong antigens that can be demonstrated by the resistance to the growth of the tumors when tested by transplantation to specifically immunized syngeneic animals. However, these tumor antigens are very difficult to demonstrate by tests done in the animal of origin [9].

In the case of the virally induced tumors, the usual explanation for the difficulty in immunization of the animal of origin is that such animals are partially tolerant to the virus which forms an intrinsic part of the tumor antigen. Tolerance to the carcinogen is apparently not a factor in chemically induced tumors, but the difficulty of immunizing the animal of origin is, at least in part, thought to be caused by the well-known immunodepressive activity of the carcinogen [10].

In contrast to the chemically and virally induced tumors, sporadic tumors of unknown etiology in rodents, are usually weakly, if not completely, nonimmunogenic by similar tests. That they may indeed possess a slight immunogenicity is suggested by the fact that in the oft cited paper by Hewitt [11], 7 of 7 such tumors, tested by specific immunization, showed a small increased growth apparently produced by the immunization procedure [11].

Yet another situation in which the immunogenicity of a tumor is relatively difficult to demonstrate results from the “sneaking through” phenomenon: a tiny amount of transplanted antigenic tumor often grows well when a larger inoculum would have failed [12]. It may be important to note that the animal of origin of any tumor has perforce been exposed to what may be an analogous phenomenon. Each tumor originates from a single or at best a tiny clump of cells and thus must “sneak through” in that original animal. This manner of exposure to antigen apparently produces some form of partial tolerance to the tumor antigens, while the tumor remains demonstrably immunogenic if tested in a different animal. The prevalence of at least a partial tolerance to the tumor's antigens in the animal of origin seems to further assure that tumors will usually exhibit in that animal only a weak immunogenicity that will thus usually lie in the stimulatory part of the IRC.

## 5. Clinical Implications

Parenthetically, the Lappé data suggest that the immune reaction worked almost entirely upon the longevity of the papillomas; the number of paps becoming malignant was a function of the number of paps remaining at risk and was a constant proportion of those paps surviving into successive time intervals [5]. Kreider et al. also found, in somewhat analogous experiments with papilloma induction in rats by the Shope papilloma virus, that altering the host's immune capacities altered primarily the longevity, not the incidence, of paps [13]. This curious result is overtly similar to the appearance of mammary tumors in females of Dr H.B. Andervont's milk-agent-infected C3H mouse colony; after the ninth month mammary tumors appeared, at successive monthly time intervals, in a constant proportion of the females remaining at risk [14]. Also, other data have shown that, while the annual cancer incidence of each individual varies depending upon the number of years of exposure to tobacco smoke, in the ex-smoker, the risk of cancer tends to become a constant in each successive year after he or she stopped smoking [15–17]. Seemingly, it may be a common property of many types of carcinoma to arise, in each successive time interval after the initiating events, in a constant small proportion of those benign lesions that have not yet regressed and are still available for malignant progression. 

The existence of tumor stimulation rather than surveillance following tumor initiation may help to explain some otherwise perplexing observations. One of the most dramatic of these, consistent with the hormetic shape of the IRC (Figure 1) and the immune stimulation hypothesis, is the behavior of Kaposi's sarcoma. This lesion is common in AIDS patients, but it often “flares” as the immune mechanism recovers during therapy [18]. The most likely interpretation appears to be that the tumor grows best (is stimulated) when the patient's immune capacity is low, but not too low. If the sarcomas were to grow better in AIDS patients because of a lack of immune inhibition, they would not be expected to flare, as they do during a transitory period of partial immune recovery.

Further clinical evidence that may support the idea of immune stimulation of tumor growth in the primary tumor bearer has recently been presented by Roscigno et al.: removal of tumor-negative lymph nodes, in a very large cohort (412 patients) with upper-tract urothelial carcinomas, significantly improved the prognosis [19]. This finding suggests that this system lies to the left of “c” on the IRC and may have resulted from the removal of tumor-stimulatory lymphoid elements rather than, or in addition to, the more conventional explanation that undetected microscopic metastases were removed along with the lymph nodes.

A large number of other correlates of the immune stimulation hypothesis have been discussed elsewhere [8, 20], among which is the observation that immunodepression has been reported to reduce rather than increase the incidences of both human breast [21] and rectal [22] cancers. Immune surveillance has no explanation for this behavior, but according to the immune stimulation hypothesis, these tumors are probably so weakly immunogenic that they usually fall to the left of “c”. In that stimulatory region of the IRC, further reduction of the immune reaction would lead to less tumor stimulation. Thus, as may be suggested by both human breast and rectal carcinoma incidences, a reduced immune capability may sometimes be less, rather than more, conducive to oncogenesis. The fact remains that the incidences of most human tumors, probably as a result of tolerance as well as of a relative paucity of antigenicity, have been reported to be little affected by immunodepression of the host [22], albeit that this observation has been questioned [23].

## 6. Mechanism of Stimulation

The mechanism of immunostimulation is still, I think, undetermined. Perhaps the simplest way to think of the whole phenomenon of hormesis may be to recognize the likelihood that every living tissue has probably evolved to be capable of a variety of compensatory defenses against any likely toxin (the immune response in the present case) and that such anti-toxic devices may often overcompensate to a small extent (hormesis is typically a phenomenon of small extent [24]). The fact that some human cancers are apparently helped to grow by immunodepression (skin and lymphoid tumors) while others (breast and rectal tumors) are perhaps relatively inhibited suggests the hypothesis that most oncogenesis probably occurs on the IRC either immediately to the right or to the left of “c” in the stimulatory part of the IRC curve.
